# InSAR-observed surface deformation in New Mexico’s Permian Basin shows threats and opportunities presented by leaky injection wells

**DOI:** 10.1038/s41598-023-42696-9

**Published:** 2023-10-12

**Authors:** E. J. Graves, A. Rinehart, R. Grapenthin, M. F. Angarita, J. Grigg

**Affiliations:** 1 Department of Geosciences, University of Alaska Fairbanks, Fairbanks, AK USA; 2 Department of Earth and Environmental Sciences, New Mexico Institute of Mining and Technology, Socorro, NM USA; 3 Former Subsurface Geologist, New Mexico Bureau of Geology and Mineral Resources, New Mexico Institute of Mining and Technology, Socorro, NM USA

**Keywords:** Hydrology, Natural hazards, Solid Earth sciences

## Abstract

Knowledge of aquifer dynamics, including groundwater storage changes, is key to effective groundwater resource and reservoir management. Resolving and accurate modeling of these processes requires knowledge of subsurface poroelastic properties and lateral heterogeneity within units of interest. Computationally demanding methods for determining lateral heterogeneity in poroelastic properties exist but remain difficult to practically employ. The InSAR-based detection of uplift over a New Mexico well with a casing breach provides an opportunity to determine poroelastic properties using a tractable 2D analytical plane strain solution for surface uplift created by a pressurized reservoir with overburden. Using a Bayesian inversion framework, we calculate poroelastic properties under deep (depth of well-screen) and shallow (depth of well-breach) conditions. We find that shallow injection is necessary to produce the observed deformation. However, pressure-varying forward solutions for uplift are required to reproduce the temporal evolution of deformation. For this we use realistic shallow poroelastic properties and well dynamics, which reflect the evolving injection conditions at the well breach as the casing further erodes. Analysis of individual interferograms or InSAR time series may provide insights into shallow subsurface heterogeneity or anomalous injection conditions at operating wells more rapidly than scheduled field inspections.

## Introduction

Large-scale deformation caused by groundwater pumping and oil and gas production has been identified using InSAR^1–7^. These observations help us understand injection impacts on hazards such as aquifer pollution, halite dissolution and sinkhole formation^8, 9^, as well as the potential for induced seismicity^10^. They are also important for groundwater resource and reservoir management, monitoring, and sustainability as surface deformation provides insight into the temporal and spatial dynamics of aquifer storage capacity and a way to observe unrecoverable aquifer storage loss^3, 4, 11, 12^.

Understanding subsurface properties and their lateral heterogeneity is another key factor in the management and monitoring of reservoirs. In most cases, aquifers are developed through trial-and-error drilling with limited geophysical surveys, though this is changing with evolving technologies^13, 14^. The lateral boundaries of productive regions often can only be defined at the level of ‘between this well and that well’. Productive fluvial and alluvial formations that commonly house freshwater aquifers are especially heterogeneous, with lateral boundaries sometimes appearing nearly randomly and at short length scales^13, 14^. The ability to map hydraulic properties without expensive, indirect geophysics or prohibitively expensive drilling programs has remained a grand challenge in hydrology from the first uses of groundwater^15, 16^. Efforts towards using InSAR to characterize lateral heterogeneity include the joint analysis of InSAR and hydraulic head measurements taken at observation wells^17–21^ or production wells^12^ to derive hydromechanical properties (i.e., skeletal storativity, specific storage, and compressibility) and the identification of new faults disrupting groundwater flow^22^. However, the analysis of sub-kilometer scale deformation in wellfields to gain insight into poroelastic properties has been largely overlooked.

Alghamdi et al.^23^ use a computationally expensive Bayesian inversion method for InSAR observations over a wellsite in Nevada to find variations in lateral permeability, inferring up to ~ 17,000 permeability parameters. The authors use a 3-D geomechanical model with over 320,000 pressure, displacement, and fluid flux state variables. The complexity and computational cost of running such an extensive inversion, however, makes implementation prohibitive for many practical applications.

**Figure 1:**
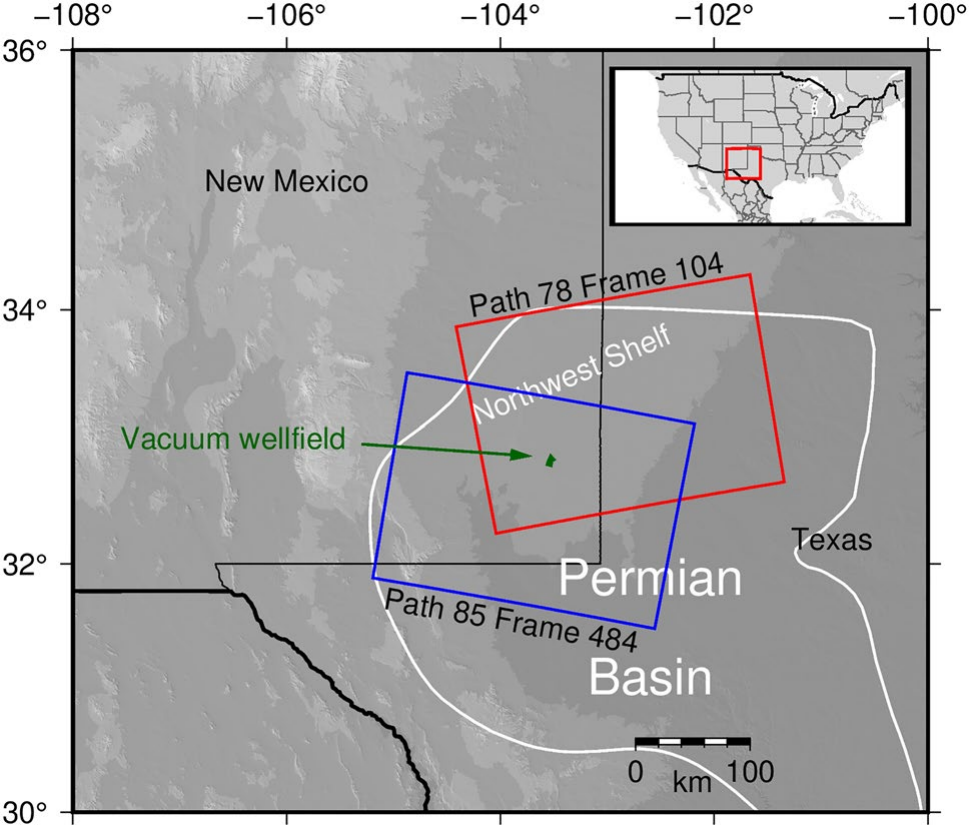
Sentinel-1 SAR frame locations. Outlined in red, the location of the ascending Sentinel-1 frame overlies the Vacuum wellfield and covers a portion of the Permian Basin. We outline the descending frame in blue.

Here, using InSAR time series, we resolve an injection plume that propagates outward from a well in southeastern New Mexico’s Vacuum wellfield in the Permian Basin (Fig. 1) starting roughly 7 months prior to any anomalous well condition reports. Zheng et al.^24^ describe one example of such sub-kilometer scale deformation at a wastewater injection well in West Texas attributed to casing failure and/or sealing problems and propose utilizing InSAR as an indirect leakage monitoring method. Our findings support this proposed method and illustrate its effectiveness compared to scheduled inspections.

Our resolved time series of deformation reveals an initially nearly circular deformation pattern that over time develops a linear asymmetry, which we believe is part of a subsurface lateral heterogeneity in shallow formations. To understand which formation parameters are most reflected in deformation in a homogeneous formation, we determine poroelastic properties of the subsurface from the data utilizing a simple 2-layer analytical model coupling a 1D groundwater flow equation with a plane strain solution for surface uplift due to a pressurized reservoir with overburden. This essentially controlled shallow injection experiment demonstrates the value of high-resolution remotely sensed surface deformation time series in imaging and characterizing lateral heterogeneities either in structure or lithology of groundwater bearing formations with direct implications on the modeling of groundwater storage and aquifer dynamics. Importantly, it suggests that deformation data can be used in some cases to determine aquifer properties – hydraulic diffusivity, formation thickness, lateral boundaries – without the need for a dense monitoring well network. This also provides a method for well field monitoring that can be more effective and timely in its response than traditional manual inspections.

## Data and methods

### InSAR analysis

We utilize synthetic aperture radar observations collected by the European Space Agency’s Sentinel-1 A/B mission^25^ on ascending path 78 frame 104 and descending path 85 frame 484 from October 2016 through April 2019 (Fig. 1). Digital elevation models for terrain corrections were created using NASA Shuttle Radar Topography Mission (SRTM-1) elevation data with 30 m resolution^26^. Utilizing the open source InSAR processing software GMTSAR^27^ to generate 2 pass interferograms, we set a temporal baseline of 800 days and a perpendicular baseline of 200 m for choosing interferometric pairs (Table S1). The arid and sparsely vegetated southeastern New Mexico retains higher coherence levels over longer temporal baselines than regions with a larger percentage of vegetation cover and/or snowfall, allowing us to create often highly coherent multi-year pairs.

All interferograms are created with the same reference acquisition (17 Dec. 2016 for the ascending path; 11 Dec. 2016 for the descending path) and many display high coherence and minimal short wavelength atmospheric noise (Fig. S1). Because of the observed low variation of atmospheric noise on the sub-km scale we investigate, we do not employ modeled atmospheric corrections. From the high coherence interferograms, we created a fixed-reference time series. To unwrap the modulo 2π radian observations of the interferograms into line-of-sight (LOS) displacements, we use the Statistical-cost, Network-flow Algorithm for Phase Unwrapping (SNAPHU)^28^. We then cut the phase images to a local scale and removed a best-fitting bi-linear ramp function to reduce long-wavelength background noise, such as tropospheric and ionospheric effects^29^. To compare the InSAR observations to geomechanical modeling results, we decompose the ascending and descending data sets spanning similar time periods into near-vertical and near-east deformation fields^30^.

### Groundwater and geomechanical modeling

We follow the methods outlined by Wangen et al.^31^ to create a 2D analytical model of the injection conditions and lithology at wellsite American Petroleum Institute (API) No. 3002524312. Modeling reservoir pressure with a 1D, transient solution to the groundwater flow equation for an isotropic and homogeneous formation, we set no-flow boundaries at the top and bottom of the aquifer, an infinite right-hand domain, and a fixed pressure boundary on the left-hand side (the location of the wellbore). We assume a plane-strain approximation for modeling geomechanics and couple the equations via standard poroelasticity through the Biot coefficient to tie effective stress and overpressure to each other. We assume the formation elastically deforms much faster than the pressure wave propagation which allows a one-way coupling from the groundwater flow equation to the plane strain deformation solution.

This plane strain solution, derived from a Fourier decomposition of the reservoir overpressure, determines the surface uplift over a pressurized reservoir and an additional layer of overburden. This simple two-layer model contains a reservoir with a thickness, *h*_1_, and an overburden of thickness *h*_2_. The lateral domain extends from -*L* to *L*. Boundary conditions include the absence of vertical displacement at the base of the reservoir, the overburden surface experiences zero normal stress, and the pressure distribution, *p(x)*, is symmetric about the wellbore or z-axis at x = 0 and reaches zero before the domain ends at |x|= L.

Following Wangen et al.^31^, we determine the surface uplift, $w$, at location x and time t from the combined Fourier series terms for a given initial pressure, *p*_*0*_, using Eq. (1):

1 $$w\left( {x,t} \right) = \frac{{\alpha h_{1} p_{u} \left( {x,t} \right)}}{{\left( {\Lambda + 2G} \right)}}$$

where $\alpha$ is the Biot coefficient, Λ is the Lameé-parameter, and *G* is the shear modulus. Because the pressure distribution $p_{u} \left( {x,t} \right)$ from injection is an even function, it can be represented by a cosine-function Fourier series:

2 $$p_{u} \left( {x,t} \right) = \frac{{a_{0} }}{2} + \mathop \sum \limits_{n = 1}^{\infty } f\left( {k_{n} h_{1} ,k_{n} h_{2} } \right)a_{n} cos\left( {k_{n} x} \right).$$

where the function *f*, a dimensionless value between 0 and 1, represents the amplitude at the surface which depends on the wavenumber, $k$, and the thicknesses of *h*_1_ and *h*_2_ written as

3 $$f\left( {kh_{1} ,kh_{2} } \right) = \frac{1}{{kh_{1} }}\left( {cosh\left( {kh_{2} } \right)tanh\left( {kh_{1} + kh_{2} } \right) - sinh\left( {kh_{2} } \right)} \right).$$

We find the Fourier coefficients, *a*_*n*_, for the pressure distribution by integrating Eq. (4)

4 $$a_{n} = \frac{2}{L}\mathop \smallint \limits_{0}^{L} p\left( {x,t} \right)cos\left( {n\pi x/L} \right)dx,n = 0,1,2,...$$

where *p(x,t)* represents the overpressure at time *t* and location x given an initial fluid pressure *p*_*0*_ and hydraulic diffusivity *D*. Assuming uniform hydraulic diffusivity and solving the groundwater conservation equation in 2D, we approximate the pressure wave propagation with.

5 $$\begin{array}{*{20}c} {p\left( {x,t} \right) = p_{0} erfc ({\frac{x}{{2\sqrt {Dt} }}}}) \\ \end{array} ,$$

where the erfc or complementary error function denotes the area under the tails of a Gaussian probability density function^32^.

We model two separate conditions at the wellsite to account for the well’s history according to the State of New Mexico Oil Conservation Division (NM-OCD) which provides details of operational conditions, injection / production, and maintenance on individual wellsites including those located within the Vacuum wellfield. From their reports we know Texaco Inc. began drilling API well No. 3002524312 in December 1972, and reached a total depth of 1463 m before production started the following January 1973^33^. Conversion of the well from oil production to water injection occurred in the early 1980’s. An injection profile survey reported on 14 Aug. 2017, resulted in the shut in of the well following the identification of a casing leak between 256 and 265 m. All attempts to repair the well failed, and after once again finding material from the surrounding Dewey Lake Formation redbeds in the wellbore, the well was plugged and by 01 Sep. 2017 the site was abandoned. To resolve the source of deformation, we create a model for the intended deep injection into the San Andres formation at 1463 m depth, and another for shallow injection occurring at the well breach between 256 and 265 m.

The Guadalupian-aged Grayburg and San Andres formations form the primarily carbonate reservoir of the Vacuum wellfield. High-angle, low-displacement faults horizontally compartmentalize the reservoir^34^. Largely dolomite, the reservoir also includes sections of impermeable anhydrite cementation and very low permeability thin, interbedded dolomitic siltstones^34^, which also may compartmentalize the reservoir. Overlying Guadalupian formations of dolomite and anhydrite include secondary shale and sandstone layers. Younger formations are predominantly composed of dolomite, anhydrite, sandstone, and halite^34^. The youngest formation, the Late Permian Dewey Lake Formation, consists of anhydrite cemented fine sandstone and siltstone^35^. Schiel^36^ attributes the geomorphologic features and sedimentology of the formation to deposition in an ephemeral fluvial system featuring sheet floods, and broad, shallow channels up to 2 m thick that stack and crosscut one another. Above the Dewey Lake Formation lies the Triassic-aged Santa Rosa Formation (Dockum Group), a complex body of sandstones with channels up to 30 m thick that have been found to be permeable across the region^37^. Other channel sand bodies have been found in the Dockum Group above the basal Santa Rosa Formation as well^38^ and are anecdotally described as ‘pockets of water’; however, due to poor water quality in this region, the Dewey Lake Formation sand bodies are relatively unexplored.

### Inverse modeling

We formulate the forward model described above as an inverse problem to determine model parameter values from observed deformation. We utilize the VMOD inversion framework^39^ that implements Bayesian inversions via a Markov Chain Monte Carlo (MCMC) method to infer posterior probability density distributions of values for free model parameters: depth to reservoir/overburden thickness, width of reservoir, borehole pressure, diffusivity, and Young’s modulus. We begin with uniform prior distributions using parameter bounds accounting for the well geometry and lithology of the reservoir and overburden during deep injection (Table 1). The prior distribution is uniform to characterize the uncertainty of the parameter values while the a priori values are chosen within the established parameter bounds as the first set of parameter values from which we first determine the likelihood of creating the observed uplift.

**Table 1:** Parameter distribution ranges—the ranges for the deep injection uniform prior distributions are limited by the intended well operating conditions and the lithology surrounding the well from the reservoir through the overburden.

Model	Parameter bound	Reservoir thickness (h1, m)	Overburden thickness (h2, m)	Borehole pressure (P0, MPa)	Diffusivity (D, m^2^/s)	Young's modulus (E, GPa)
Deep injection	Lower	1	1000	1	1.00E−10	1
	Upper	1000	10,000	100	1.00E−01	100
Shallow injection	Lower	1	1	1	1.00E−04	1
	Upper	1000	1000	100	1.00E−01	100
Pressure varying forward model	Lower	15	100	10	1.00E−03	5
	Upper	50	300	70	1.00E−02	15

For the shallow injection models, the prior uniform distributions were updated to reflect shallow injection conditions in the well where the lithology changes due to the presence of the Dewey Lake Formation. The parameter bounds for the pressure varying forward models are limited to the realistic values for the reservoir geometry and lithology at shallow depths which narrows the bounds of the model parameters.

We invert near-vertical uplift profiles for the time steps creating a single posterior distribution. The profile location (A to A’; see Results in section "InSAR") was selected away from asymmetric portions of deformation to adhere to the symmetry assumption of the model (see Results). In the inversion we used profiles through 210 days (t6, see Fig. 5) and we determined a single borehole pressure applied throughout the simulation period.

The inversion uses 3,100,000 steps with a burn-in period of the first 100,000 steps that are discarded to remove biases due to the choice of a priori parameter values. We then sample every 1,000 steps to avoid collecting strongly correlated samples. This produces 3,000 posterior distribution samples that determine the parameter posterior probability density distributions.

**Figure 2:**
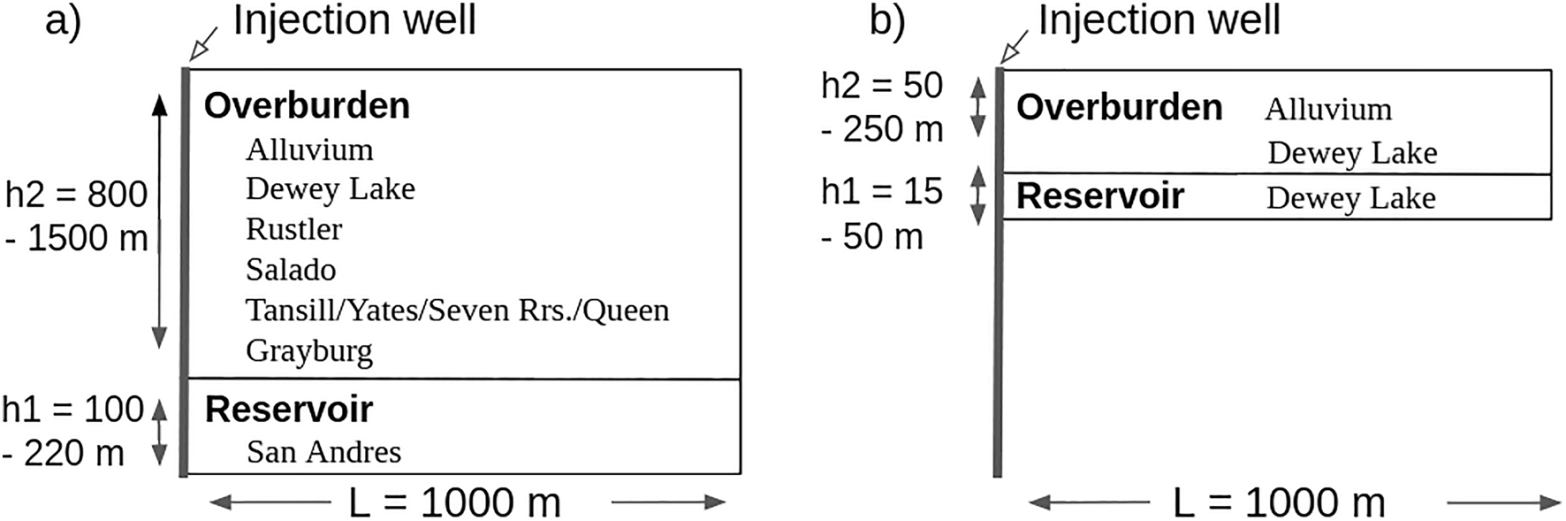
Models of the injection well conditions are calculated with a Biot coefficient of 0.7 and a Poisson’s ratio of 0.2. (**a**) Deep Injection Model Schematic—In this layout, the injection of the fluid occurs around the perforated wellbore interval. The overburden consists of the overlying formations. (**b**) Shallow Injection Model Schematic—Injection of the fluid occurs much shallower in the wellbore with less overburden.

In order to recreate the intended operating conditions at the wellsite, we use a two-layer model consisting of the pressurized Grayburg/San Andres reservoir and the overburden of largely carbonate rock (Fig. 2a). The lateral domain extends to 1000 m. For the pressure distribution calculation, we utilize the first 100 Fourier coefficients *a*_*n*_. Reasonable values for the Biot coefficient ($\alpha$ = 0.7) and the Poisson’s ratio $\nu$ = 0.2) are chosen, and conversions from $\nu$ and Young’s modulus, *E,* determine values for $\Lambda$ and *G*.

To determine the magnitude of uplift in our shallow injection model we utilize the previously described method with the same foundational assumptions. We change the uniform prior distribution ranges (Table 1) to model injection through the shallow well casing breach. The redbed Dewey Lake Formation becomes the pressurized reservoir at the breach and the overburden consists of sandstones, siltstones, and alluvium (Fig. 2b).

## Results

### InSAR

**Figure 3:**
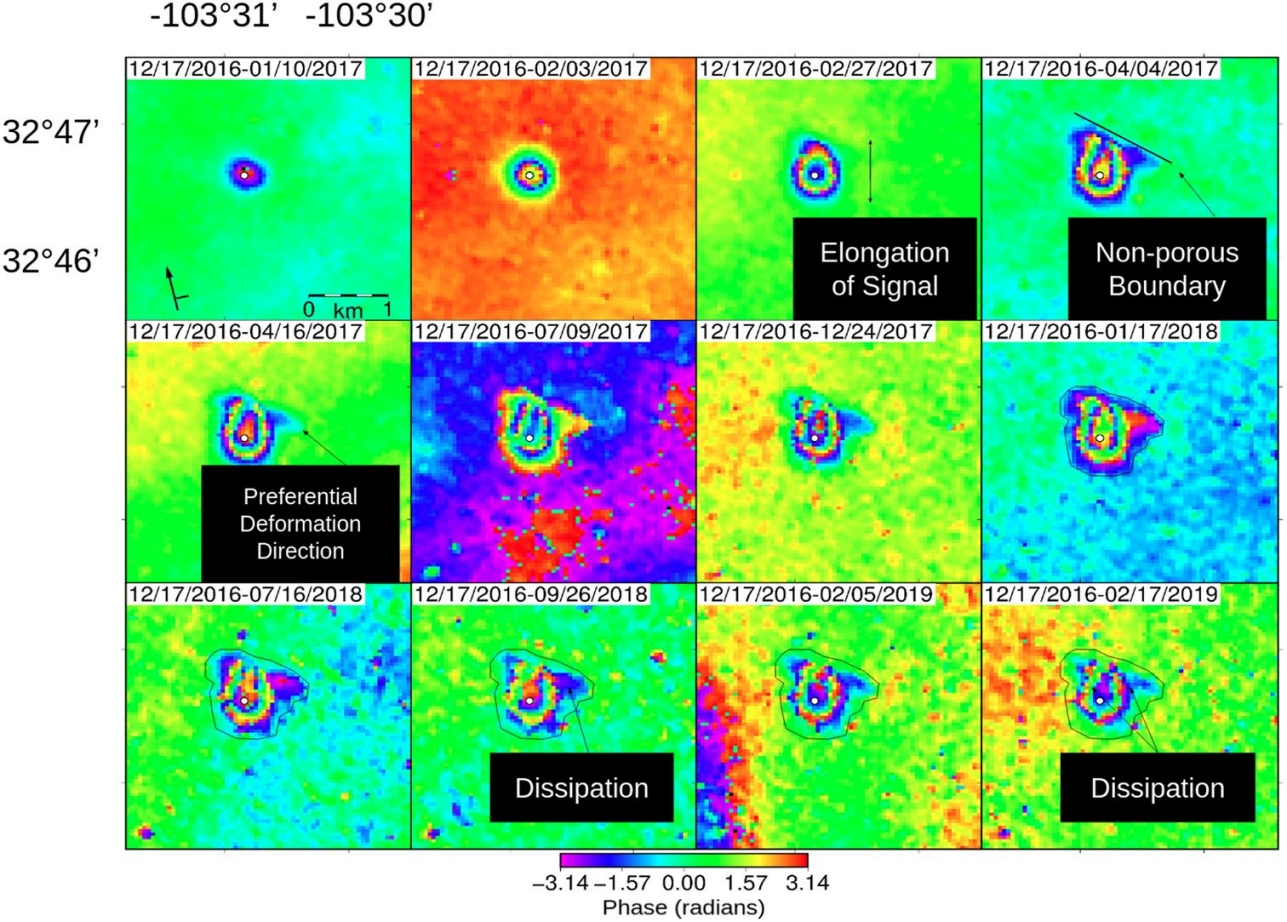
API No. 3002524312 Wellsite fixed reference image time series of two pass wrapped interferograms from December 2016 until February 2019 on ascending path 78 frame 104. An anomalous signal lies over an EOR injection well (white circle) and grows over time developing a direction of preferential deformation. Maximum extent of deformation occurs in panel 8, where the signal is outlined in black, which is replotted in the following panels where the deformation dissipates.

The ascending fixed-reference time series spanning 17 Dec. 2016 through 17 Feb. 2019 (Fig. 3) shows a distinct sub-kilometer deformation signal (Fig. S1) overlying a known enhanced oil recovery (EOR) wellsite, API No. 3002524312. The initially radially symmetric signal evolves substantially over the more than 2 years of observation. Starting as a ~ 0.15 km circle of deformation in January 2017, the signal develops into a ~ 0.6 km dome in the wrapped phase around the wellsite by early February 2017. We note the northward elongation of the signal early in the InSAR series (26 Feb. 2017) while the southern portion of the signal remains radially symmetric. A linear northeast edge forms by 4 Apr. 2017, highlighting part of a subsurface feature longer than a kilometer striking northwest-southeast. A lateral barrier to flow (i.e., a sharp decrease in permeability) within the formation could result in the observed feature, whatever its source. Deformation develops along the linear trend preferentially to the southeast creating an asymmetrical horseshoe shape where half of the signal south of the well retains its symmetry.

**Figure 4:**
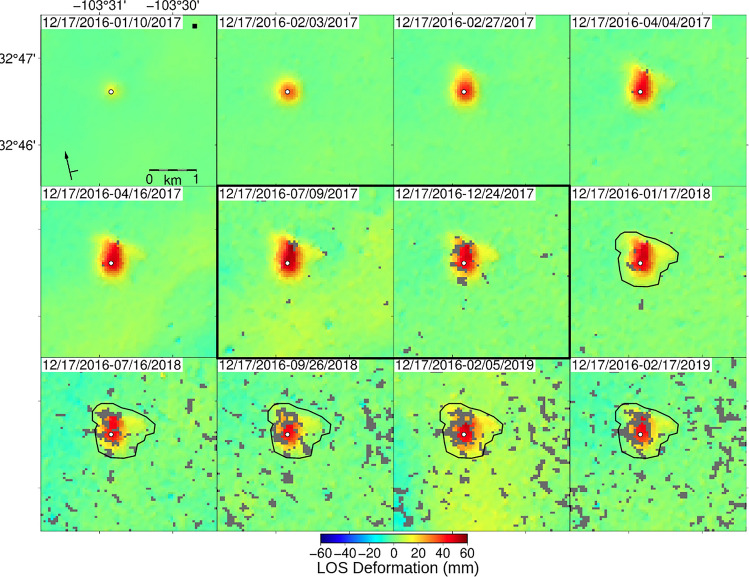
Ascending path line-of-sight (LOS) deformation at API No. 3002524312—The LOS deformation at the chosen modeling times illustrates the complex temporal evolution of the signal over more than two years. Initial deformation of roughly 14 mm, shown in panel 1, begins radially symmetric about the wellsite before deforming preferentially to the north (panel 2) and then moving along a northwest-southeast trend (panel 3 and 4) before reaching a maximum vertical deformation of ~ 66 mm on 09 Jul. 2017 (panel 6). The location of the reference pixel is indicated in black on panel 1. Abandonment of the wellsite occurred in September 2017 between the two interferograms bordered in black (panel 6 and 7); decay of the vertical deformation begins by the first acquisition following the plugging of the well (panel 7).

**Figure 5:**
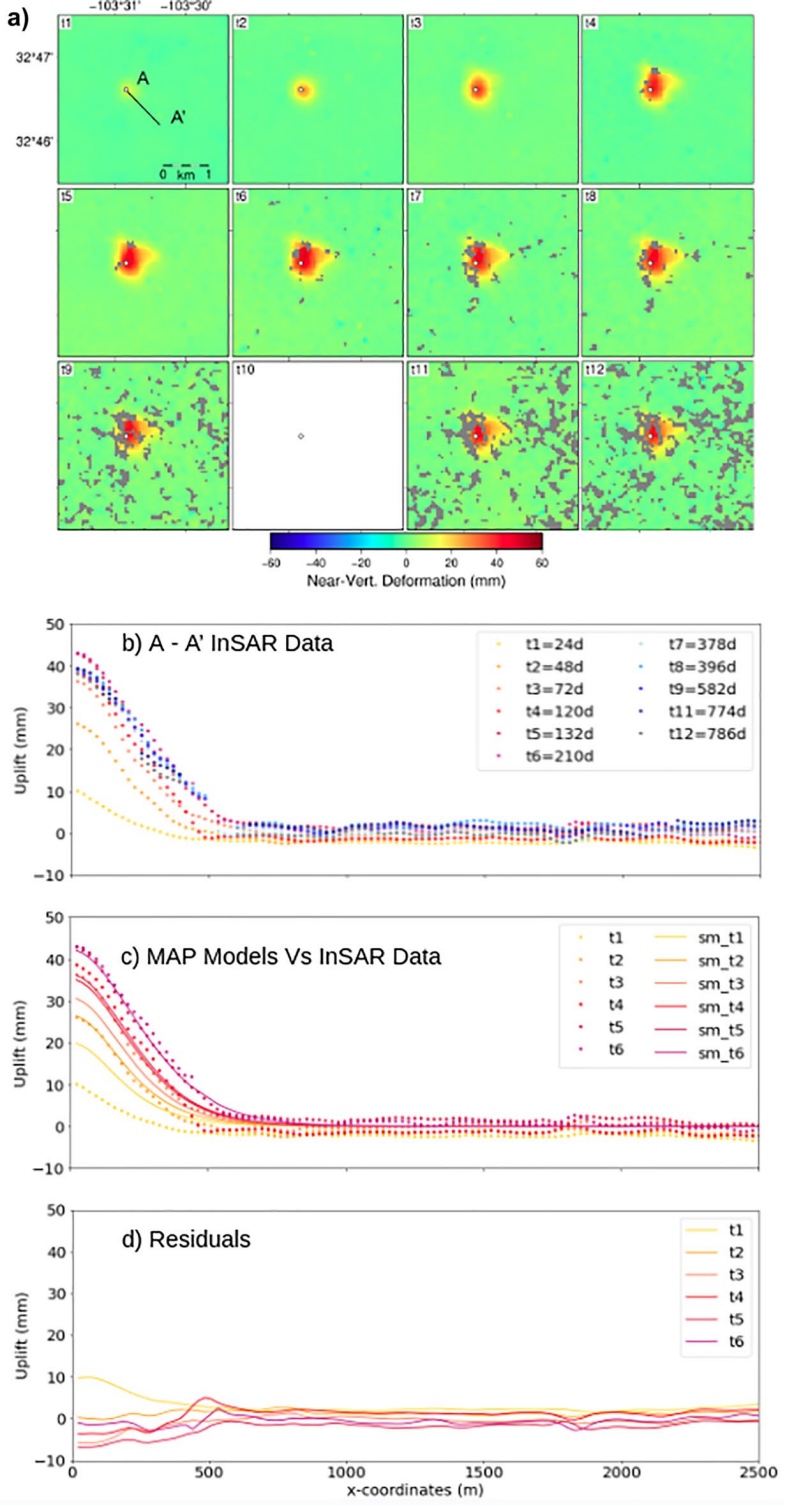
Near-vertical deformation at API No. 3002524312 shown in map-view (panel 1 shows the location of **A** to **A**’) and along profile A to A’. Panel t10 is left blank because time differences between ascending and descending path repeat interferograms exceeded 50 days; decomposition into near-vertical would not faithfully represent the process. Hence, to illustrate this lack of data and allow comparison with approximate times of other panels in phase images we leave this space. (**b**) The A to A’ near-vertical deformation profile is plotted by color according to time (hotter colors during rapid expanse; cooler colors during slowly decreasing extent) and show a rapid increase in vertical deformation up to 210 days followed by a slow decrease in the deformation. (**c**) The forward models (sm) of the shallow injection inversion’s best-fitting or maximum a posteriori parameter values are plotted (solid lines) for comparison to the near-vertical InSAR deformation values during the period of injection. (**d**) Model residuals show that we achieve the best fits at times t2 and t6, and the worst fit is associated with time t1, focussed in the near field of the well.

This asymmetry is consistent with image well theory (see Discussion in section "Discussion"). Image well theory and superposition predict that the portion of the pressure plume going away from the barrier (to the south of the well) would be least affected by the barrier, if at all. These observations provide insight into the lateral heterogeneity of the unit the fluids are migrating through at depth. This signal asymmetry and the inferred subsurface heterogeneity are confirmed in the unwrapped line-of-sight (Fig. 4) and near-vertical (Fig. 5) deformation fields.

Maximum extent of deformation, ~ 1 km in the radially symmetric region and ~ 1.2 km perpendicular to the observed linear trend, at the wellsite occurred on 17 Jan. 2018 (Figs. 3, 4, outlined in black) at 396 days, and subsequent interferograms show the gradual dissipation of the deformation area while the signal retains its shape. The coherence of the images decreases through the time series leading to its truncation at the final phase image in February 2019. The phase images for the descending frame (Fig. S2) show similar, albeit somewhat noisier, observations suggesting that the dominant signal of this process is in the vertical direction.

Unwrapping the panels in Fig. 3 yields the ascending line-of-sight (LOS) deformation time series (Fig. 4), which displays the same temporal evolution of the signal’s footprint as described above in the wrapped phase images; however, we can more readily observe the magnitude of deformation from the LOS figure. At the first time step in the series, spanning 24 days (10 Jan. 2017), we observe ~ 14 mm of LOS deformation concentrated near the wellsite with a satellite look vector of ~ 347° and an average incidence angle of 35°. By 108 days (04 Apr. 2017) the LOS deformation has spread further outward from the wellsite. Maximum observed LOS deformation of 55.3 mm is reached at 204 days (09 Jul. 2017), but maximum lateral spread of the plume is observed at 396 days (17 Jan. 2018). Deformation slowly contracts vertically and horizontally after 204 and 396 days, with the last observation at 792 days (17 Feb. 2019) showing ~ 46 mm of LOS deformation. The LOS deformation maps for the descending frame are included in the supplemental section (Fig. S3).

The decomposition of ascending and descending LOS deformation produces near-vertical (Fig. 5) and near-east (Fig. S4) deformation time series. The near-vertical deformation (Fig. 5) accounts for the majority of the observed LOS deformation. We do not have data for time t10 as the temporal span between LOS images would be over 50 days, which we consider too long to assume similar LOS signals in both viewing geometries, therefore the panel is left white, maintaining the graphical association between Figs. 3, 4, 5. Coherence remains high until times t9, t11, and t12 (582, 774, and 786 days). Maximum deformation occurs at time t6, at roughly 210 days, totalling about 50 mm. Deformation then dissipates as observed in phase and LOS space in Figs. 3 and 4. For modeling we extract near-vertical values along profile A to A’ from the radially symmetric portion of the signal (Fig. 5).

### Geomechanical modeling

Using reservoir and overburden properties for the region, we model the resultant surface uplift for two injection scenarios (at intended injection depth and at the shallow casing breach) and compare the values of uplift to observed InSAR uplift profiles (Fig. 5).

#### A model of surface deformation during deep injection at a wellsite

**Figure 6:**
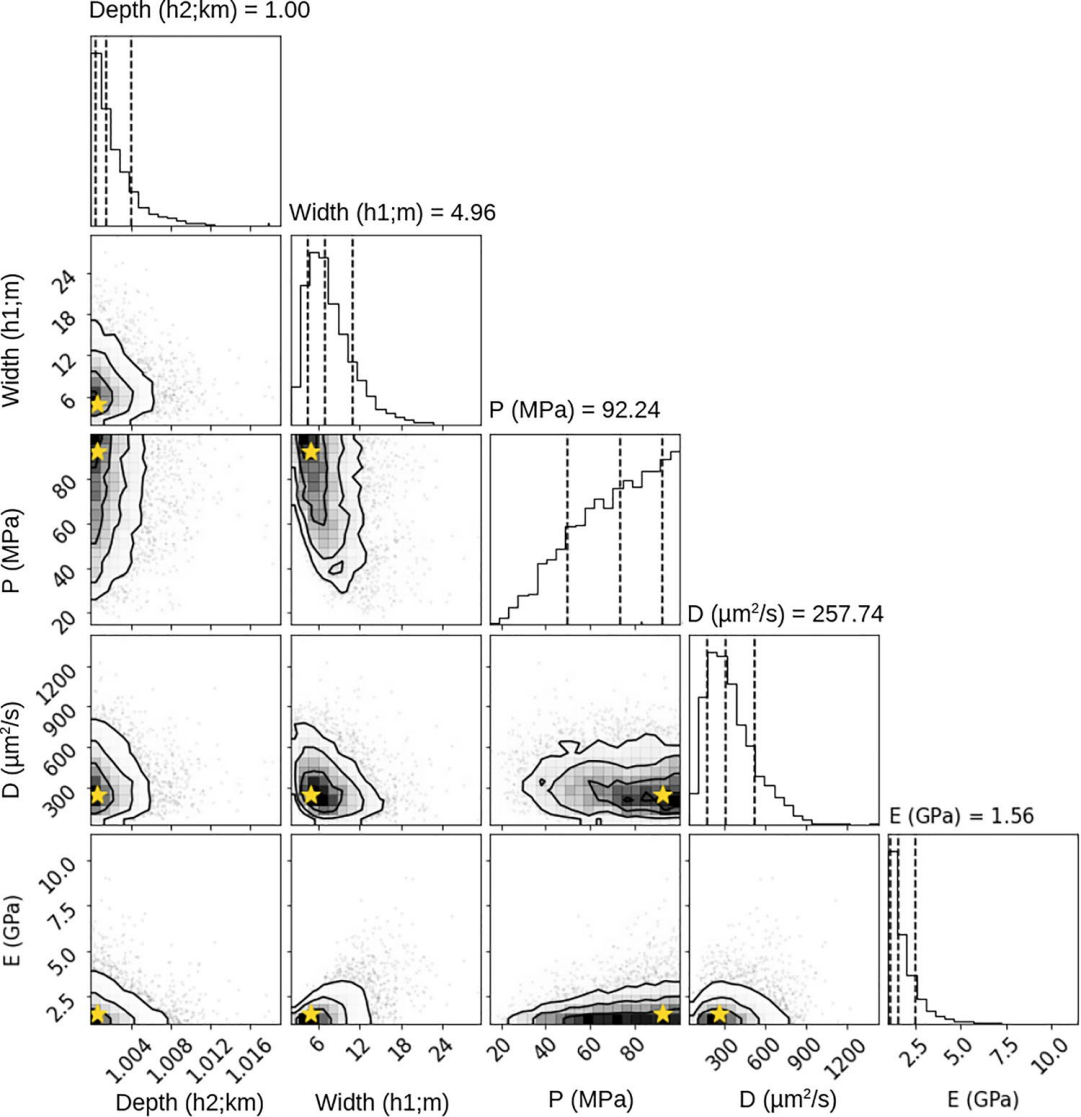
Deep Injection Inversion. The results for our inversion when enforcing a prior uniform distribution that includes a limit, 1000 m, for how shallow injection can be modeled, illustrates posterior distributions that cluster towards the parameter bounds we imposed. The median values (central dotted line in graphs) and MAP values (stars) are not realistic for the known conditions at the wellsite. The reservoir would be thin and very close to the surface compared to the perforated interval within the well bore given these MAP values. Young's modulus is lower than expected; however the diffusivity is reasonable for the given lithology. The borehole pressure MAP value is significantly higher than the reported operational conditions. The first standard deviation is shown as dotted lines on either side of the median value.

Setting the depth of injection to the range where perforations were made in the lowest section on the well with an upper bound of 1000 m in the prior uniform distribution, we invert the deformation observations for the model parameters reservoir depth and thickness, borehole pressure, diffusivity, and Young’s modulus. The posterior distributions in Fig. 6 show that deep injection does not capture the characteristics of the data, resulting in poorly fitting models (Figs. S5, S6) with parameter value distributions hugging the parameter bounds. The posterior probability density distributions show that there is little correlation between model parameters.

The maximum a posteriori (MAP) value for depth is just below the imposed 1000 m limit, while the reservoir thickness of ~ 5 m and a Young’s modulus of ~ 2 GPa are not realistic values for characterizing the deep injection conditions and lithology. The MAP borehole pressure of ~ 92 MPa is also significantly above the reported operational conditions (10—15 MPa). The value for diffusivity (~ 2.6 × 10^–4^ m^2^/s) is physically reasonable for the rock type. The deformation resulting from the unrealistic MAP values for the poroelastic parameters significantly underestimates the observed deformation reaching a maximum of ~ 20 mm (Fig. S5), while forward models using realistic parameter values predict sub-mm scale deformation (Fig S6).

#### A model of surface deformation at a casing breached wellsite

**Figure 7:**
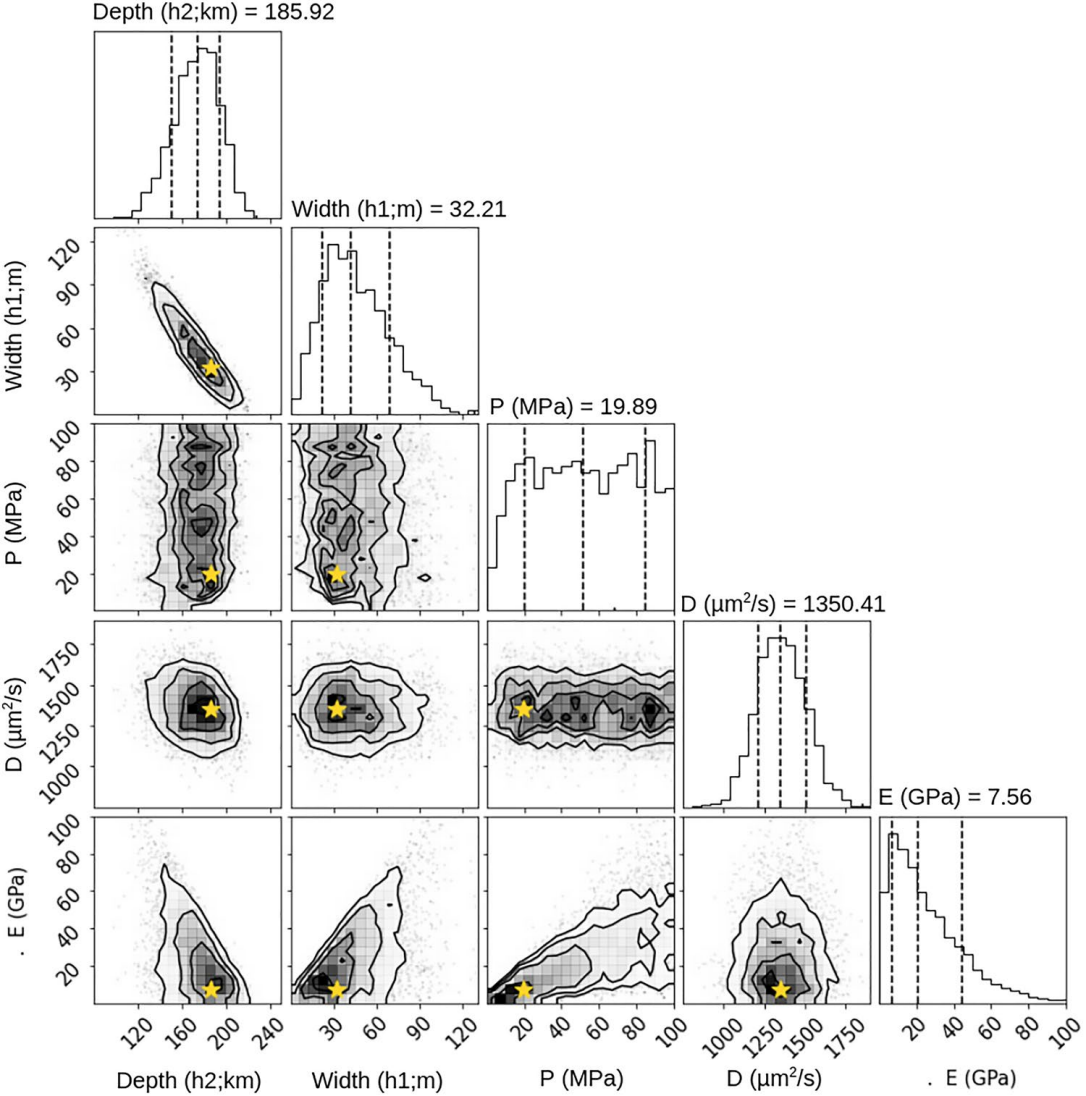
Shallow injection depth inversion. Like Fig. 6, we can observe trends in the probability density functions from the inversion allowing for shallow injection. The most noticeable is the trade off between depth and width of the reservoir, where the width decreases with depth. The diffusivity probability is limited to a narrow band of values, while the borehole pressure is poorly constrained. MAP values (stars) for the reservoir location indicate injection occurs shallow in the well bore at ~ 186 m with a thickness of ~ 32 m. The borehole pressure, diffusivity, and Young’s modulus MAP values are within reason for the given lithology. The first standard deviation is shown as dotted lines on either side of the median value (central dotted line).

Historical well injection data from the NM-OCD documents the presence of a casing breach somewhere between 256 and 265 m^33^. If the injected EOR fluids were not fully injected into the intended horizon, and instead at least partially migrated through the casing breach into the much shallower formations we might expect larger uplift and a better fit to the observations. Switching the model setup to the schematic shown in Fig. 2b, we run the inversion to determine the posterior probability density distributions within which we determine the MAP values for the free parameters (Fig. 7); suggesting a thickness of ~ 32 m for the reservoir (h_1_) and a depth or thickness of overburden (h_2_) of ~ 186 m. The value of reservoir depth or overburden thickness is not consistent with NM-OCD reported values. MAP values for a diffusivity of ~ 1 × 10^–3^ m^2^/s and a Young’s modulus of ~ 8 GPa are reasonable values for the known geology, but a borehole pressure of ~ 20 MPa falls slightly outside of the reported operational range of values. The surface deformation for time t1-t6 given the MAP parameter values is plotted in Fig. 5c, where we can observe well-fitting models at times t2 and t6. The modeled maximum value of vertical deformation at time t6 is ~ 42 mm, slightly less than the observed near-vertical of ~ 50 mm. Figure 5c also shows a large residual for the first time step, t1.

We observe a trade off between values of depth and reservoir width where a deeper reservoir corresponds to a smaller reservoir thickness which allows for higher pressures in the reservoir. A narrow posterior distribution for diffusivity shows sensitivity for determination when using our methods, while a broad distribution for the Young’s modulus and borehole pressure reveals that they are not as well determined. Given the generally non-Gaussian distributions, we do not report the formal standard deviations of the parameter value distributions.

## Discussion

InSAR observations over southeastern New Mexico that span more than three years, the limit of coherence in this Sentinel-1 frame, document the temporal evolution of an injection-driven ground deformation signal. The study of individual interferograms (Fig. 3) provides insight beyond the location of deformation and in fact allows for the direct observation of a portion of a local lateral subsurface heterogeneity, confirmed in the near-vertical transformed observations (Fig. 5), and likely extending beyond the imaged length of the InSAR-illuminated lineation. A purely homogeneous subsurface would produce a radially symmetric signal (i.e., a circular pattern of vertical deformation with the well at the center in the phase image, see modeled deformation in Fig. S7). The elongation of the northward propagating InSAR signal along the NW–SE direction (Figs. 3, 4, 5) is consistent with what would be expected from the presence of an impermeable barrier, creating the effect of an image well in the observed signal^40^. Consistent with the image well solution, a clear linear feature develops along this barrier and deformation is noticeably more extensive in the southeast. We expect fluid migration to be altered by changes in permeability/lateral heterogeneity in the formation; therefore, preferential deformation to the southeast of this feature allows us to infer higher permeability in this region. The observed changes in poroelastic properties could be attributed to the stacked and cross-cutting fluvial channels of the Dewey Lake Formation.

Determining poroelastic properties for the region surrounding the wellsite becomes difficult when the assumed homogeneous overburden in our model is known to be heterogeneous (see lithological descriptions in section "Groundwater and geomechanical modeling"). We model fluid flow without a stress-dependent permeability but estimate the normal poroelastic coupling using the Biot coefficient based on rock type. Significant coupling between the mean stress and immediate (months to years) stress history with permeability is not expected considering the following conditions. One, the fractured limestone of the San Andres Formation should show the greatest mean stress dependence on permeability^41^; however, the formation depth limits the effect on the signal even with very large (1–3 orders of magnitude) changes in permeability. Two, the Dewey Lake Formation sand bodies, which are Santa Rosa Sandstone-equivalent sand bodies (Dockum Group), where we believe the leak occurred, generally have little clay and have undergone extensive lithification^42–46^, limiting the coupling. Three, the mean stress dependence of permeability in lithified materials at the depths of the San Andres Formation and Dockum Group generally results in permeability changes on the order of 10% (mean stress changes of 10 s MPa) which would exceed the ‘fracture gradient’^47^ and the casing leak would have likely been larger and apparent at the surface.

Because the polar orbit of the Sentinel-1 satellites results in low sensitivity to north–south deformation, we expect that some of this deformation has been mapped into the near-vertical signal. However, based on the near-east signal amplitudes (Fig. S4), we expect this error to be small. Nevertheless, this assumption likely results in overestimation of the magnitude of the observed uplift and consequently affects the values for the poroelastic properties determined during modeling, making them upper bounds, likely also accounting for some of the residuals in our shallow injection inverse model (Fig. 5d).

Our models of deep injection using realistic parameter values, which show no significant deformation and therefore cannot explain the observations and do not help in the characterization of the subsurface. However, our simple Bayesian inversion for shallow injection provides a relatively fixed MAP value for diffusivity and Young’s modulus, but shows significantly less sensitivity to borehole pressure. In addition to determining the hydraulic diffusivity and to a lesser extent Young’s modulus, the shallow injection inversion illustrates the range of best fits for less constrained parameters from surface deformation measurements.

A range of uplift profiles from forward models of varied shallow injection conditions for the first InSAR-observed time interval shows that the solutions for the uplift observed at a single time step is non-unique with many quality model fits (Fig. S8). However, attempts to fit subsequent time steps with these same best-fitting parameters fail (Fig. S9). The forward models of the shallow injection inversion MAP values (Fig. 5c) also illustrate that quality model fits are difficult to achieve at each time step. This highlights that we are unable to determine a single set of parameters that explains the change in observed uplift over time. The assumption of constant parameter values is embedded into the forward models and thus into our inversion. While migration of the injected fluid to shallower depth is possible (albeit lacking a physical mechanism), the assumption of constant parameter values through time and space in the inversion (which uses the time varying data as input to constrain the parameters) is the most likely explanation for the unrealistic values for reservoir geometry we found (Fig. 7). The most problematic of these assumptions during a widening casing breach is the assumption that the borehole pressure remains constant.

**Figure 8:**
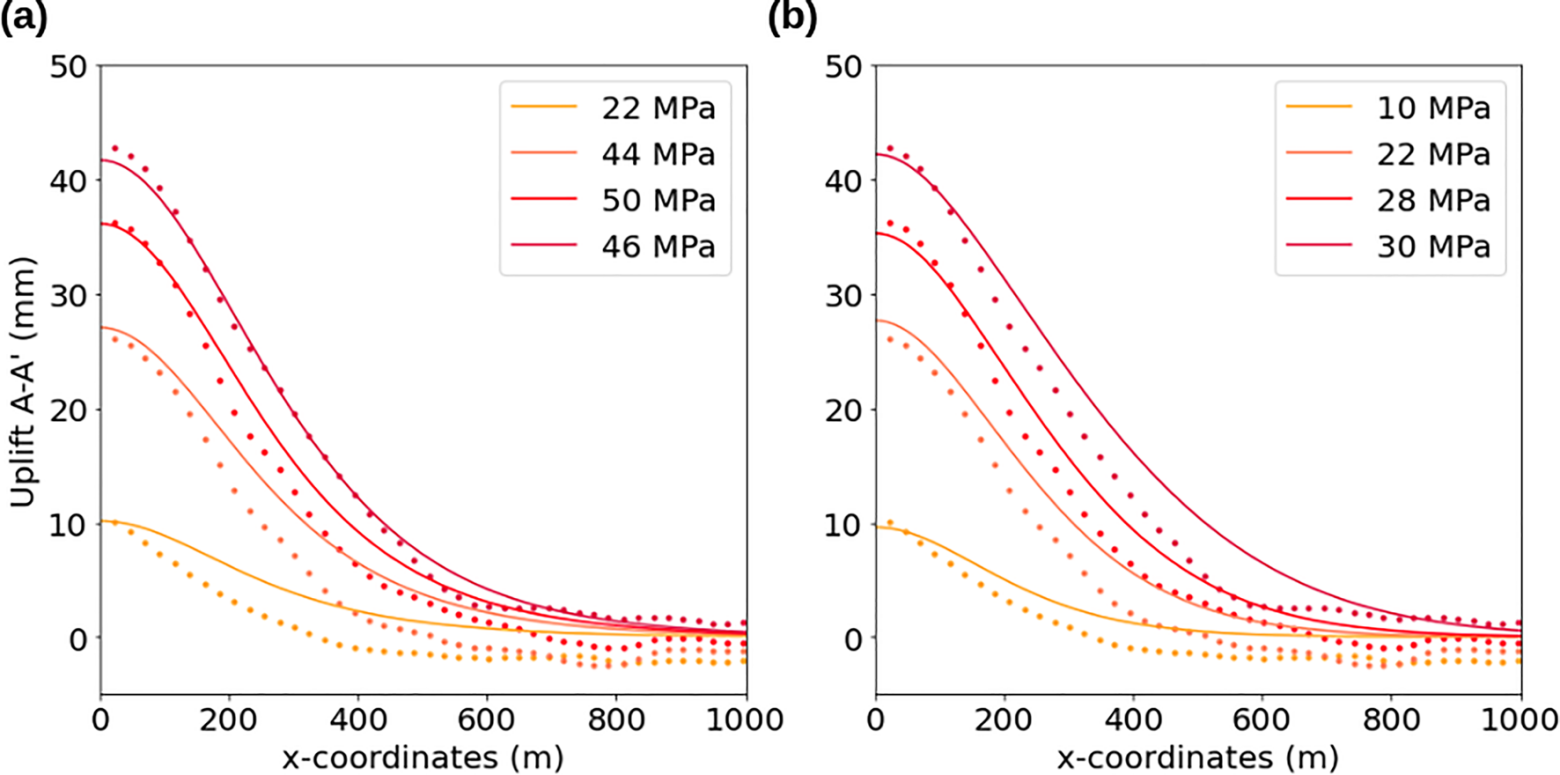
Two different pressure-varying forward models of shallow injection (solid lines) compared to InSAR uplift, along profile A—A’ (Fig. 5), for time steps t1, t2, t3, and t5 shown in warm colors (dotted lines). (**a**) High borehole pressures, low diffusivity (0.001 m^2^/s), low Young’s Modulus (8 GPa), thick overburden (250 m); The models fit well near the wellsite but over and under estimate deformation in the far field depending on the time step. (**b**) Low borehole pressures, high diffusivity (0.005 m^2^/s), higher Young’s Modulus (10 GPa), thin overburden (150 m). These models fit poorly in time step t5 compared to the previous model.

To accommodate this, we now consider the conditions of injection occurring through a widening casing breach using narrower hydraulic and poroelastic parameters bounds (Table 1). The tighter parameter bounds reflect the increased certainty in the uniform prior distribution due to the well geometry and injection pressure information provided in the well documents and the narrow posterior distributions for diffusivity and Young’s modulus from the shallow injection inversion. In other words, we explore the range of behaviors of the best-fit solutions from the Bayesian inversion. The evolution of the breach likely causes the model’s initial borehole pressure to vary over time, the most physical explanation that allows the other parameters to remain constant. We present solutions for two model geometries and elastic parameters (Fig. 8) with different constant pressures applied at the left-hand boundary (the wellbore) that fit well with the conditions at the wellsite. This allows us to examine why the inverse model is not sensitive to borehole pressure—likely because the borehole pressure varied through time.

The first model has a lower Young’s modulus and a higher overburden thickness corresponding to the breach depth, while the second model has a thinner overburden and a higher Young’s modulus. The borehole pressure in the first model is consistently higher for each time step. The overall fit is better in the first model than in the second model, especially time t5, supporting injection near the breach depth. We have a harder time fitting the far field deformation; during early time steps we slightly overestimate far field deformation while at later time steps we underestimate the values.

Our pressure-varying forward models result in smaller residuals for multiple time steps and produce solutions for our parameters that are physically realistic for the geologic setting of the wellsite. They also yield accurate injection depth determination when compared to the recorded well history. Deviations of our model results from the observed uplift away from the wellsite can be due to unmodeled subsurface heterogeneities or the assumptions of plane strain and 2D pressure diffusion break down with increasing distance from source. While we pick an EW profile that should not contain any NS motion, we cannot exclude that some horizontal motion was mapped into the near-vertical during LOS decomposition, especially as horizontal deformation increases rapidly in magnitude away from an injection site before falling off slowly.

Despite dense active injection well spacing of 0.04 km^2^^34^ in the Vacuum wellfield, we identified only one other similar signal in another southeastern New Mexico oil field during the time period analyzed in this study. This may be because the signals are difficult to find or such deformation signals are indeed scarce. Scarcity would suggest unique conditions at deforming wells, particularly since the injected fluid volumes are similar to those reported at other nearby wells (NM-OCD). Model outputs for the two cases, injection at depth and shallow injection, indicate that shallow injection would be necessary to produce the uplift observed via InSAR at the reported injection rates. This shallow injection would have been possible given the NM-OCD reported well breach. Therefore, injection of fluid and gas likely occurred at shallower depths than intended and caused the measurable expression of uplift over the wellsite similar to that observed by Kim et al.^8^.

It is worth noting that the compromised well casing or otherwise anomalous well conditions could have been reported after acquisition of the first interferometric pair in Fig. 3. However, the time between InSAR detection of deformation and the injection profile survey is ~ 7 months (reported on 14 Aug. 2017). No abnormalities were noted in an earlier annual Manual Integrity Testing (MIT) report in April 2017, when nearly 3 cm of LOS deformation had already accumulated (Fig. 4), calling into question the ability of these field tests to reliably detect well integrity issues.

During the time between casing failure and the eventual halt of well operations, water unsaturated in NaCl infiltrated the rock surrounding the breach. Had injection occurred in a salt rich formation, such as the Salado Fm. at ~ 472 m depth, about 220 m below the casing breach, dissolution of the halite could lead to karst formation and eventual collapse (e.g., Wink Sinks^8^), demonstrating the importance of timely well integrity observations.

Zheng et al.^24^ also describe an example of sub-kilometer scale deformation at a wastewater injection well in West Texas. The signal was attributed to casing failure and/or sealing problems and they proposed utilizing InSAR as an indirect leakage monitoring method. Our results show another example of InSAR’s suitability to be used for this purpose. We not only provide a unique opportunistic example comparing the modeled depth to documented well casing damage for model verification, but show how these signals can be utilized to image lateral heterogeneity and characterize the shallow subsurface and well conditions through a simple modeling approach.

## Conclusions

Using both forward and inverse models, we have shown that the InSAR observed deformation at an injection well can be attributed to the shallow injection of fluids due to a casing breach documented months after the first measurable deformation. This provides further evidence for the usefulness of InSAR-based leakage monitoring and the ability to accurately determine injection depth, while our application of Wangen et al.’s^31^ plane strain analytical model illustrates a low computational cost method of opportunistically determining poroelastic properties from the surface deformation at a wellsite. Whether shallow injection is conducted in a controlled manner, as in an aquifer test, or unintentionally as the result of mechanical failure as in our case, it can be utilized as a way to estimate hydraulic and poroelastic properties and to map local lateral formation heterogeneity.

Wangen et al.’s^31^ model can be used in a formal, relatively unconstrained Bayesian inversion to determine the effective/average diffusivity and to some degree the Young’s modulus; however, mechanical failures likely violate our assumptions of constant pressure resulting in mis-estimates of the parameters, thus showing that modeling results of well deformation have to be treated with caution. Simple pressure-varying forward models provide an approximated solution to this issue producing smaller residuals and realistic poroelastic properties and well geometries. The straightforward nature of these forward models makes analysis more approachable and time efficient for use in aquifer management and monitoring as well as oil and gas production, where early detection of casing breaches and sealing issues could prolong the life of wells, and prevent contamination or dissolution of the surrounding material and groundwater.

### Supplementary Information

Supplementary Information.

## Data Availability

All Sentinel-1 data utilized in this study is available through the Alaska Satellite Facility’s data portal (https://search.asf.alaska.edu/).
