# Author Correction: InSAR-observed surface deformation in New Mexico’s Permian Basin shows threats and opportunities presented by leaky injection wells

**DOI:** 10.1038/s41598-024-68124-0

**Published:** 2024-08-07

**Authors:** E. J. Graves, A. Rinehart, R. Grapenthin, M. F. Angarita, J. Grigg

**Affiliations:** 1 Department of Geosciences, University of Alaska Fairbanks, Fairbanks, AK USA; 2 Department of Earth and Environmental Sciences, New Mexico Institute of Mining and Technology, Socorro, NM USA; 3 Former Subsurface Geologist, New Mexico Bureau of Geology and Mineral Resources, New Mexico Institute of Mining and Technology, Socorro, NM USA

Correction to: *Scientific Reports* 10.1038/s41598-023-42696-9, published online 12 October 2023

The Acknowledgements in the original version of this Article were incomplete. The funding source for co-author M.F. Angarita was omitted. The Acknowledgements now read:

“E.J. G. and R.G. acknowledge funding through NASA grant NASA-NIP #80NSSC20K0073. M.F.A. was funded through NSF-EAR 1855126. Copernicus Sentinel data 2016–2020. Retrieved from ASF DAAC, processed by ESA. We appreciate the comments of Dr. Amoruso and two anonymous reviewers which substantially improved this manuscript. Some figures were made with GMT [48].”

The original Article has been corrected.

