# Supplementary material for: InSAR-observed surface deformation in New Mexico’s Permian Basin shows threats and opportunities presented by leaky injection wells
Source: Sci Rep. 2023 Oct 12;13:17308. doi: 10.1038/s41598-023-42696-9 (PMC10570274; doi:10.1038/s41598-023-42696-9)
Supplement: Supplementary file 1 — Supplementary Information. [file 41598_2023_42696_MOESM1_ESM.pdf]

Supporting Information for

InSAR-observed surface deformation in New Mexico's Permian Basin shows threats and opportunities presented by leaky injection wells

E. J. Graves[1], A. Rinehart[2], R. Grapenthin[1], M. F. Angarita[1], and J. Grigg[3]

[1]University of Alaska Fairbanks, Dept. of Geosciences. [2]New Mexico Institute of Mining and Technology, Dept. of Earth and Environmental Sciences. [3]New Mexico Bureau of Geology and Mineral Resources.

Contents of this file

Figures S1 to S9
Table S1

Introduction

Supplementary models referenced within the main text (Figs. S1 to S9) and a table detailing the Sentinel-1 interferometric pairs (Table S1) are provided below. The forward models are products of the methods described in the main text and differ in poroelastic properties according to the well conditions we wish to replicate.

Number	Secondary Image	Number	Secondary Image	Number	Secondary Image
1	1/10/2017	15	1/17/2018	42	1/12/2019
2	2/3/2017	16	3/6/2018	43	1/24/2019
3	2/27/2017	17	3/18/2018	44	2/5/2019
4	3/23/2017	18	3/30/2018	45	2/17/2019
5	4/4/2017	19	4/11/2018	46	3/1/2019
6	4/16/2017	20	4/23/2018		
7	4/28/2017	21	5/5/2018		
8	6/3/2017	22	5/17/2018		
9	8/2/2017	23	5/29/2018		
10	10/13/2017	24	6/10/2018		
11	11/6/2017	25	6/22/2018		
12	11/18/2017	26	7/4/2018		
13	11/30/2017	27	7/16/2018		
14	12/24/2017	28	7/28/2018		
		29	8/9/2018		
		30	8/22/2018		
		31	8/31/2018		
		32	9/14/2018		
		33	9/26/2018		
		34	10/8/2018		
		35	10/20/2018		
		36	10/31/2018		
		37	11/13/2018		
		38	11/25/20118		
		39	12/7/2018		
		40	12/19/2018		
		41	12/31/2018		

Table S1: Interferometric Pairs - The ascending fixed-reference time series has the same main reference image (12/17/2016) and the secondary images are listed here by year totalling 46 interferograms.

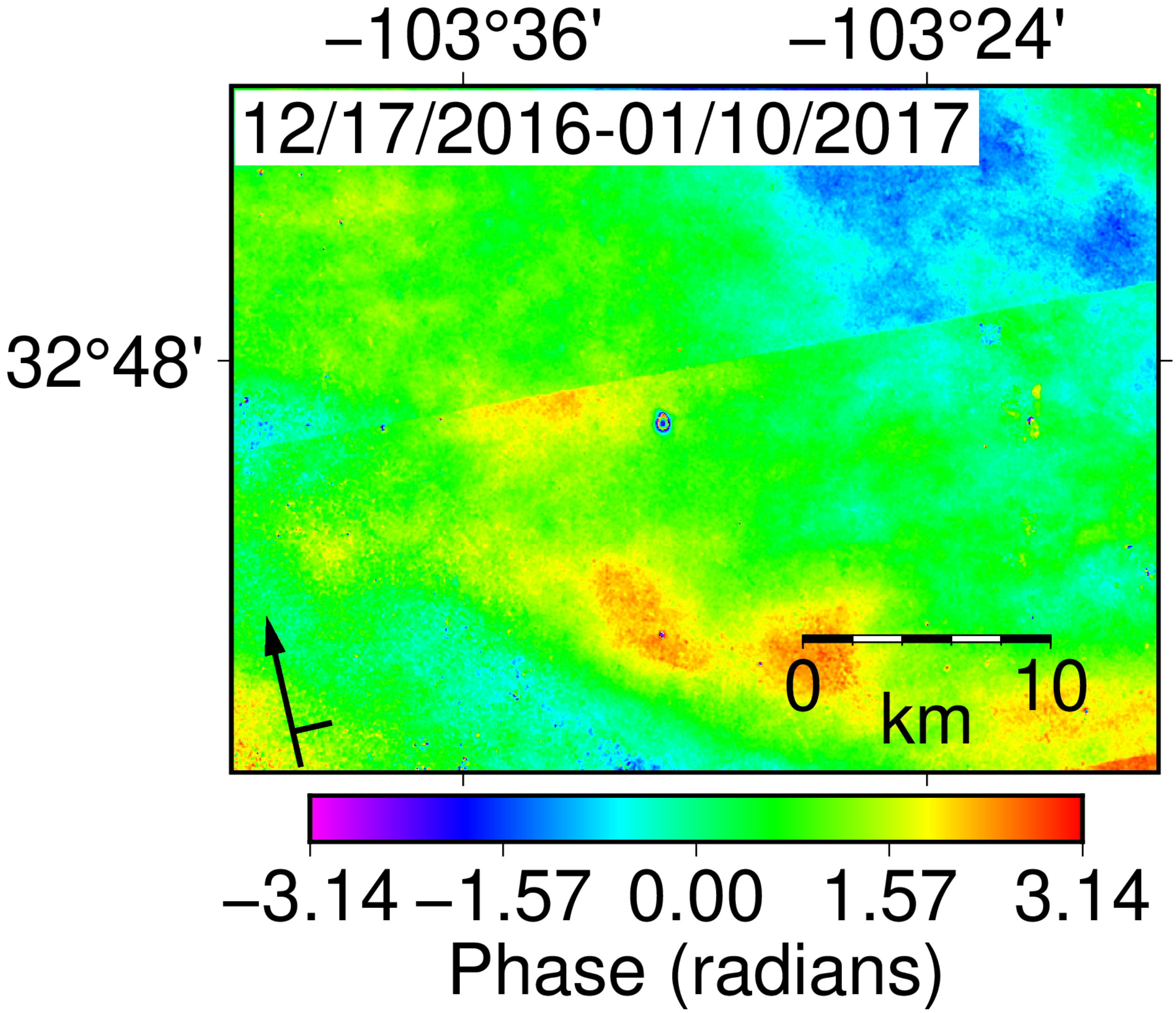

Figure S1: Regional View of Wellsite Deformation - The short-wavelength deformation associated with the wellsite stands out from the background phase values in green and differs significantly from the longer-wavelength noise within the phase values (i.e., blues and oranges).

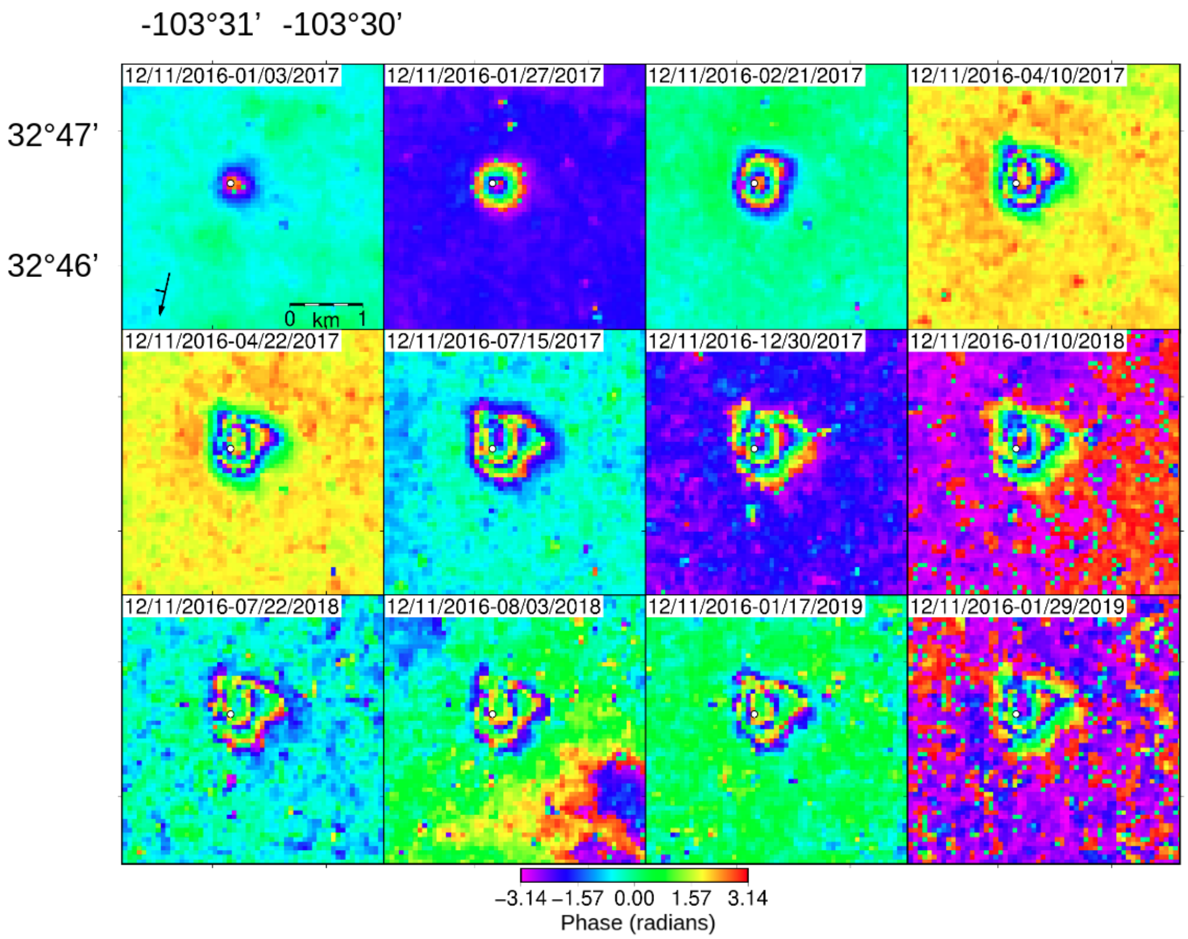

Figure S2: Descending Fixed-reference Time Series - The descending view of the deformation over well API No. 3002524312 (white circle) covers December 2016 until January 2019 on path 85 frame 484. The initially circular signal that develops an asymmetrical north-northeast border displays the same characteristics as those observed in Figure 3 on the ascending path.

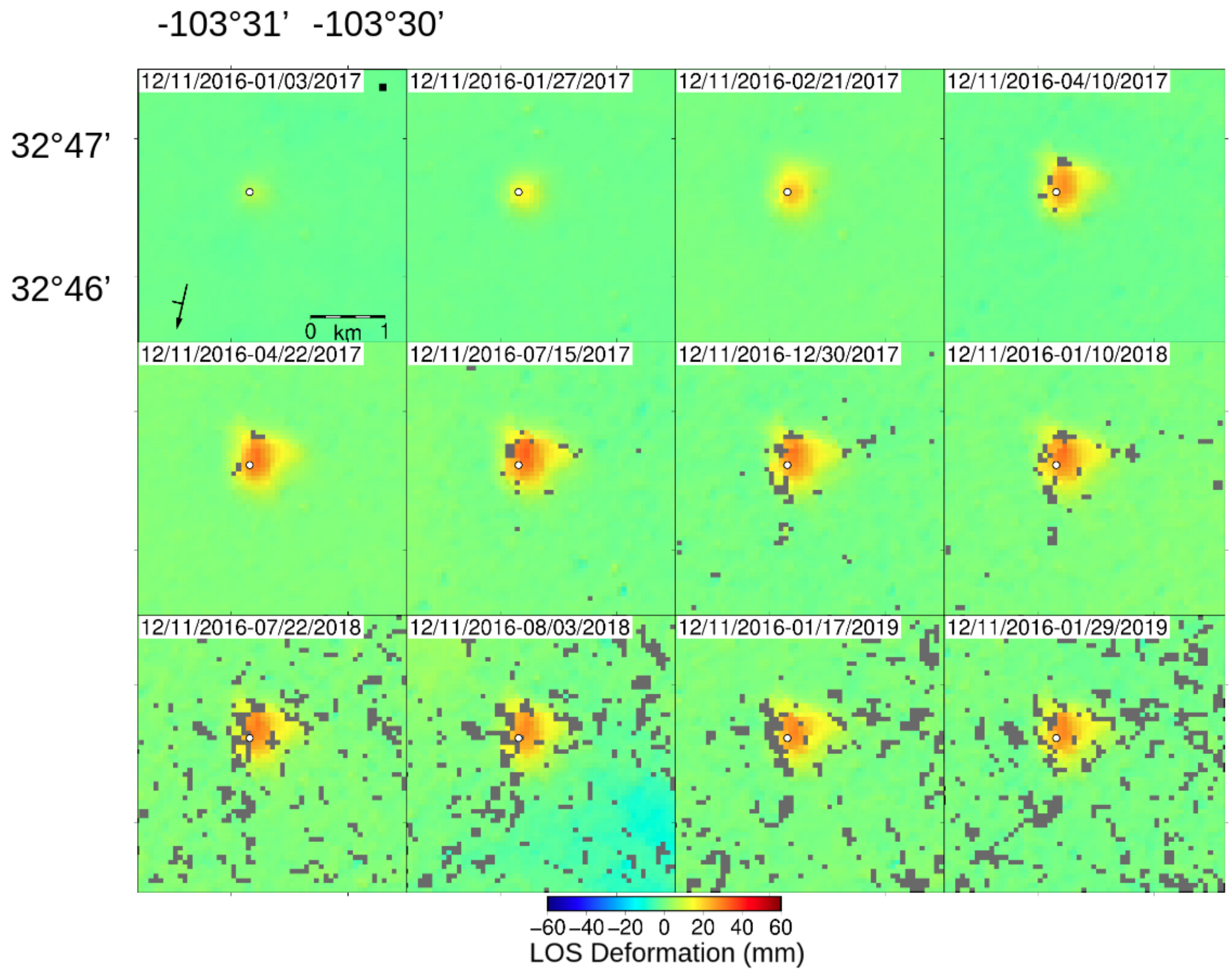

Figure S3: Descending LOS Deformation - Much like Figure S2, the descending look direction LOS deformation images display similar features over the EOR wellsite (white circle) to those observed in the ascending LOS deformation images in Figure 4. The signal initially begins symmetrical around the wellsite before developing a direction of preferential motion towards the north-northeast. The linear trend is also observed starting in panel 4 persisting until the end of the time series in January 2019.

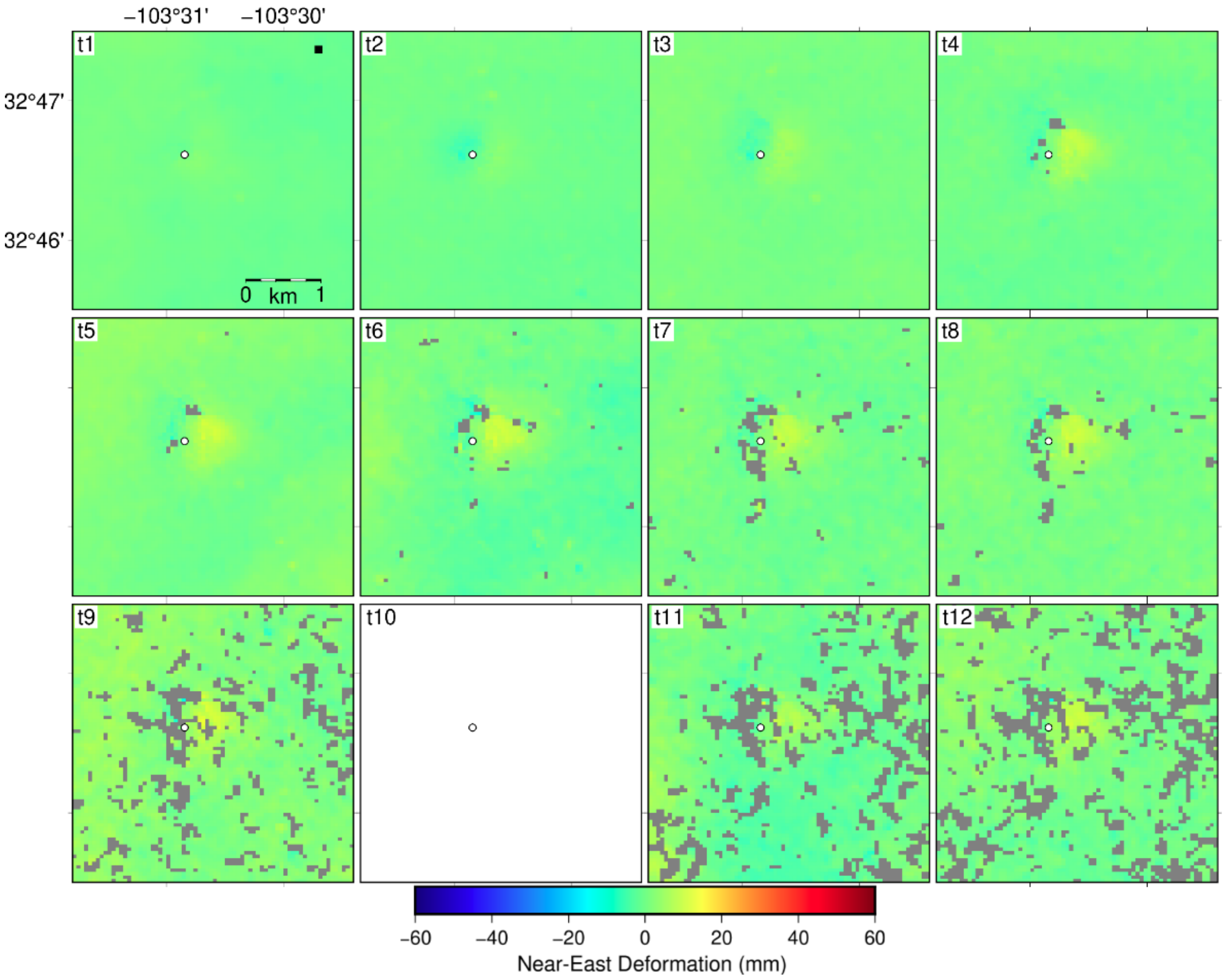

Figure S4: Near-east Wellsite Deformation - The near-east component of the decomposed LOS deformation comprises a minority of the overall observed signal. Westward motion is noted on the westward side of the wellsite (white circle) while eastward motion is present on the eastern side; observations consistent with an expanding deformation source at the wellsite.

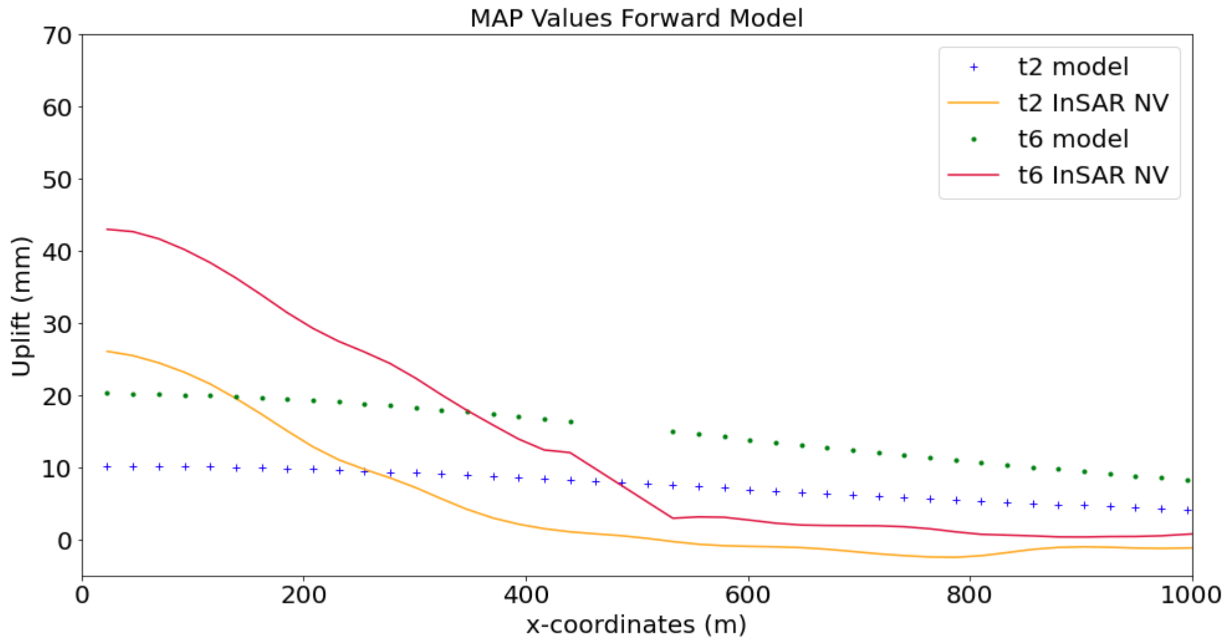

Figure S5: MAP Values Modeled Uplift for Deep Injection - Models of the uplift resulting from the Bayesian inversion MAP values at times of 48 (blue pluses) and 210 (green dots) days show ~20 mm of broad uplift, not representing the characteristics in the InSAR near-vertical (NV) data (lines).

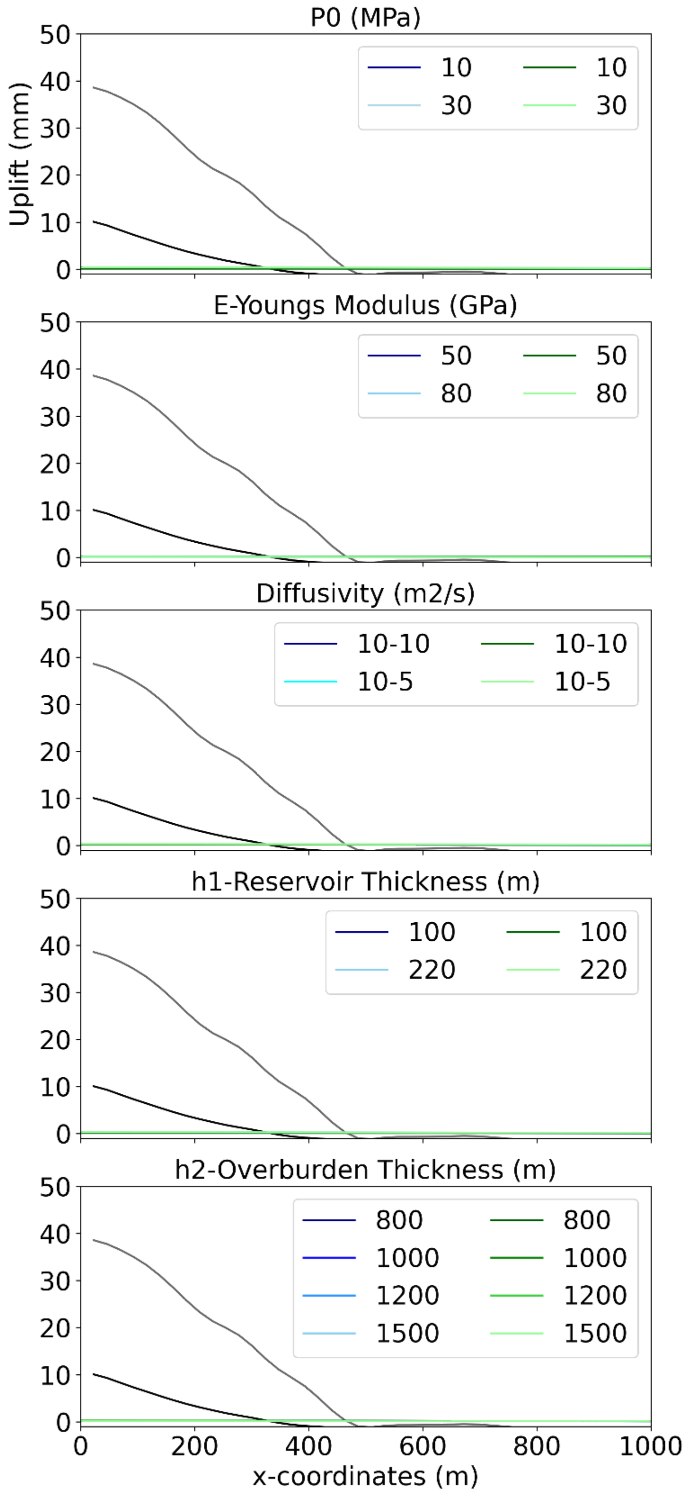

Figure S6: Deep Injection Case Forward Models - Setting the depth of injection to the range where perforations were made in the lowest section on the well, we run forward models for the first two time intervals in the uplift profiles; modeling times of 24 (blue) and 120 (green) days

were used to determine uplift while varying a single parameter. Each modeled interval, regardless of the parameter values chosen within the ranges for the given lithology and geometry, produces sub-millimeter uplift which results in the modeled deformation values overlapping each other when plotted against the InSAR observed uplift. Additional models with parameters outside of the expected operational range also results in insignificant deformation.

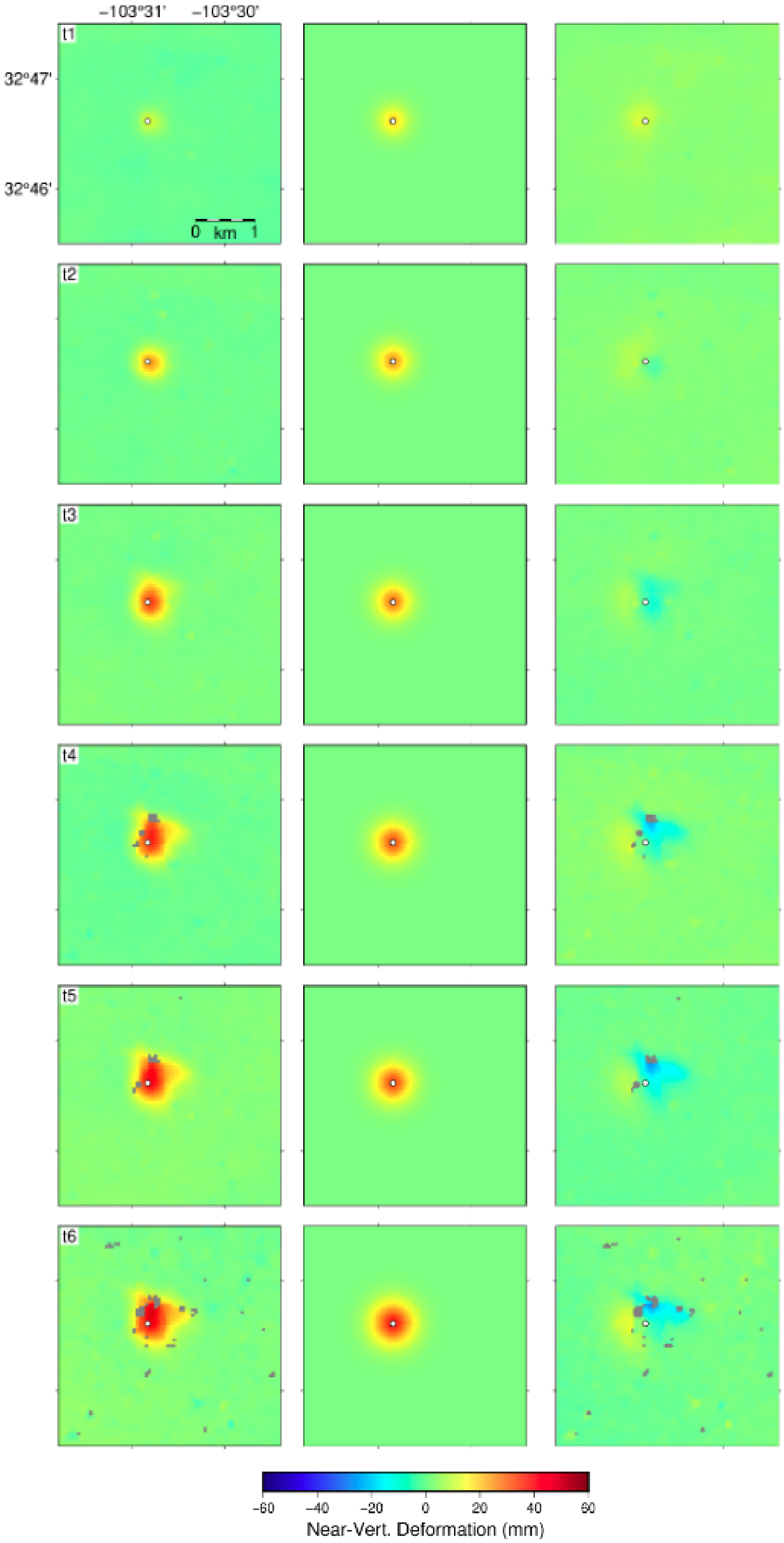

Figure S7: Shallow Inversion Model Deformation and Residuals - The first column displays the

near-vertical InSAR data for times t1-t6 (period of injection), while the second column shows the deformation field for the shallow injection inversion best-fit model of the radially symmetric portion of the surface deformation. The model residuals at each time step are shown in the third column and highlight the asymmetrical nature of the signal. A significant region of residual values is located north-northeast of the wellsite representing the elongation of the observed signal.

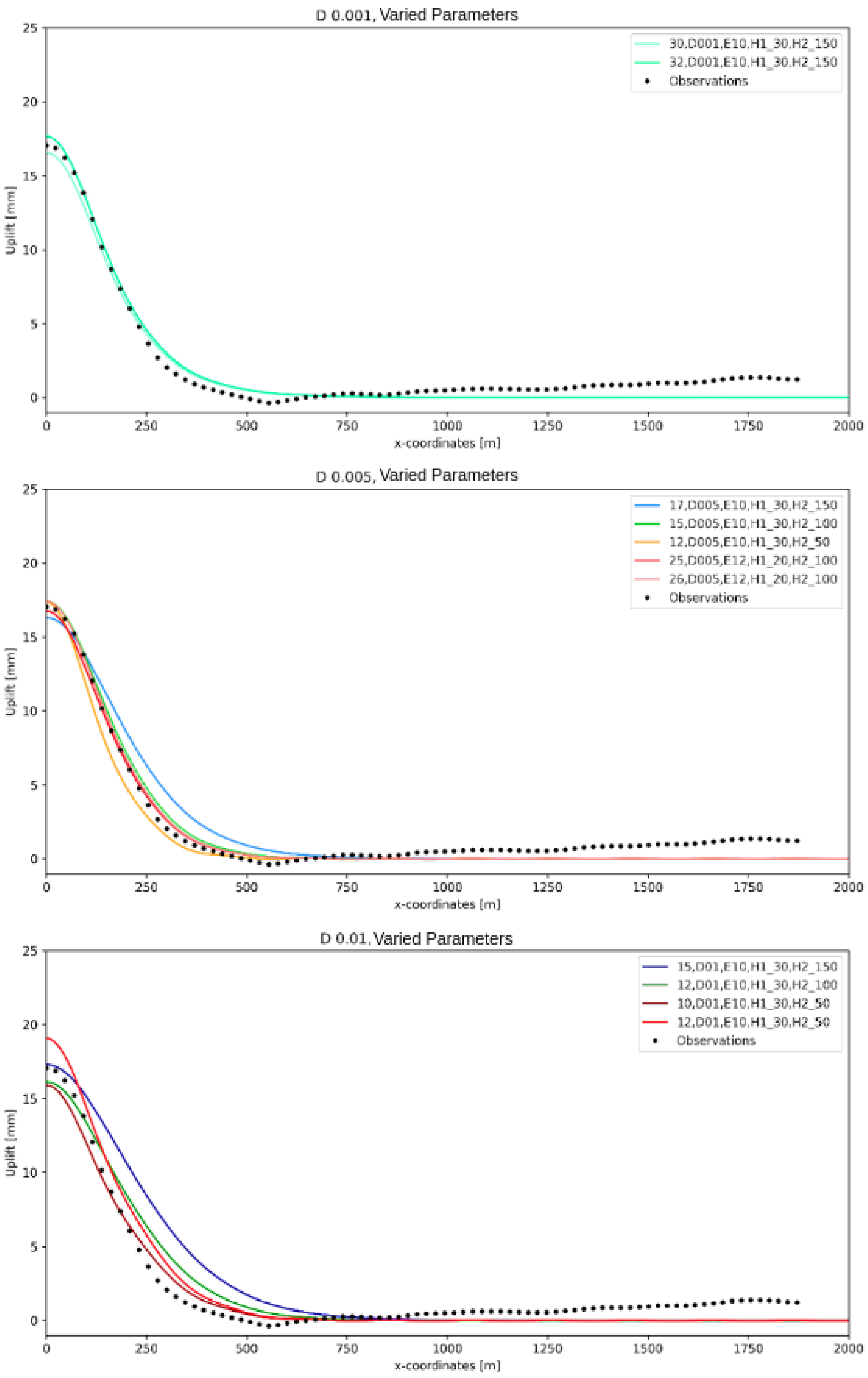

Figure S8: Non-unique Shallow Injection Forward Model Solutions compared to InSAR LOS observations along profile A-A'. When running forward models (colored lines) for the shallow

injection conditions at 24 days, we can create many combinations of model parameters that result in good fits for the InSAR observations (black dots). The abundance of models with good fits for a single time step show that solutions are not unique. The top panel is for model parameter sets with a set diffusivity of 0.001 m^2/s, the middle panel is sets with a diffusivity of 0.005 m^2/s, and the bottom panel is for sets with a diffusivity of 0.01 m^2/s. The legend lists the borehole pressure, diffusivity, Young's modulus, reservoir thickness, and reservoir depth.

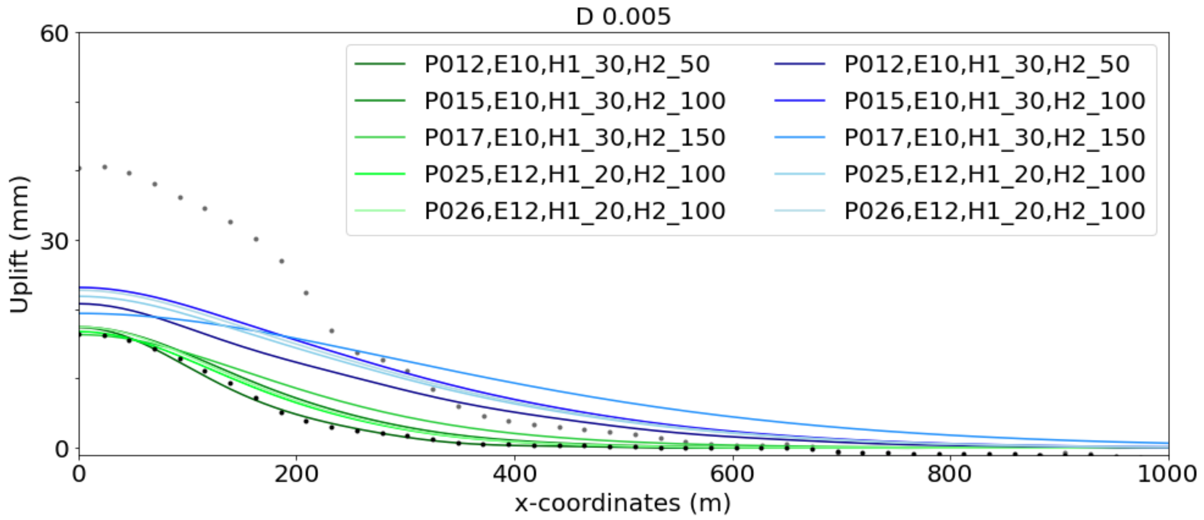

Figure S9: An Example of Ill Fitting Models with Set Initial Pressure - When initial pressure is constant over time, models at one time (green models; black dots for InSAR LOS observations) do not fit for later times (blue models; gray dots for InSAR LOS observations).
